# Tissue-Material Integration and Biostimulation Study of Collagen Acellular Matrices

**DOI:** 10.1007/s13770-021-00420-6

**Published:** 2022-03-04

**Authors:** Lindsey Alejandra Quintero Sierra, Alice Busato, Nicola Zingaretti, Anita Conti, Reetuparna Biswas, Maurizio Governa, Enrico Vigato, Pier Camillo Parodi, Paolo Bernardi, Andrea Sbarbati, Giamaica Conti

**Affiliations:** 1 Department of Neuroscience, Biomedicine and Movement Sciences, University of Verona, Strada le Grazie 8, 37134 Verona, Italy; 2 Clinic of Plastic and Reconstructive Surgery, Academic Hospital of Udine, Department of Medical Area (DAME), University of Udine, Piazzale Santa Maria della Misericordia 15, 33100 Udine, Italy; 3 Department of Plastic and Reconstructive Surgery, Azienda Ospedaliera Universitaria Integrata, Piazzale Aristide Stefani 1, 37126 Verona, Italy

**Keywords:** Acellular matrix, Capsular contracture, Adipogenic stimulation, Tissue integration

## Abstract

**Background:**

Breast reconstruction after mastectomy using silicone implants is a surgical procedure that occasionally leads to capsular contracture formation. This phenomenon constitutes an important and persistent cause of morbidity, and no successful therapies are available to date. Recently, the use of acellular membranes as a protective material for silicone prostheses has been gaining attention due to their ability to prevent this adverse outcome. For this reason, the evaluation of the tissue-material integration and the induced biostimulation by acellular membranes results crucial. Evaluation of *in vivo* tissue integration and biostimulation induced by three different natural acellular collagen membranes.

**Methods:**

Scanning electron microscopy was performed to analyse the membrane porosity and cells-biomaterial interaction *in vitro*, both in dry and wet conditions. Adipose-derived stem cells were cultured in the presence of membranes, and the colonisation capacity and differentiation potential of cells were assessed. *In vivo* tests and ex vivo analyses have been performed to evaluate dermal integration, absorption degree and biostimulation induced by the evaluated membrane.

**Results:**

Analysis performed *in vitro* on the three different acellular dermal matrices evidenced that porosity and the morphological structure of membranes influence the liquid swelling ratio, affecting the cell mobility and the colonisation capacity. Moreover, the evaluated membranes influenced in different manner the adipose derived stem cells differentiation and their survival. *In vivo* investigation indicated that the absorption degree and the fluid accumulation surrounding the implant were membrane-dependent. Finally, ex vivo analysis confirmed the membrane-dependent behavior revealing different degree of tissue integration and biostimulation, such as adipogenic stimulation.

**Conclusion:**

The physico-chemical characteristics of the membranes play a key role in the biostimulation of the cellular environment inducing the development of well-organized adipose tissue.

## Introduction

Silicone breast implants have been widely used for aesthetic and reconstructive mammoplasty [1]. The formation of the peri-prosthetic capsule (PPC) is considered part of the local reparative process against the foreign material (implant), which involves a diversity of inflammatory cells [2]. The PPC formation can become a problematic response when it contracts around the inserted material, causing hardness and deformity to the breast, known as capsular contracture (CC). According to Atlan et al. [3], collagen fibre alignment presents a significant role in CC, which means that disrupting this orientation may reduce the incidence and severity of the contracture. The differences among the surfaces of mammary implants may influence the CC. In contrast, smooth surfaces allow the proper collagen fibre alignment; the texturised ones interfere with the fibre growth protecting against the CC [3, 4].

The CC formation is one of the most commonly reported complications after implant-based breast augmentation and represents an important and persistent cause of women morbidity [5]. More than 10% of cases of CC report a noticeable and painful deformity [6]. Regrettably, there are no successful preventive therapies developed currently, and the conventional treatment requires surgical intervention for capsular remotion [5].

In recent years, researchers have been focusing on the natural tissue-derived matrices to be used as highly biocompatible and versatile scaffolds for tissue engineering applications due to native components that enhance cell migration, differentiation, and proliferation [7]. The use of acellular matrices (ACMs), of which the main element is the extra-cellular matrix (ECM) structure, has been gaining attention. They are obtained from human or animal tissues, properly treated, and deprived of any resident cell population [8].

ACMs can be used to treat difficult-to-heal wounds, deep burn closure, and as a scaffold for organ parts and tissue reconstruction due to the capacity to support host tissue cell colonisation and promote their differentiation [7, 9, 10]. ACMs healing ability is regarding their capacity to induce wound repair by amplifying the collagen secretion and deposition and adjusting the healing reparative phases [11, 12]. ACMs have been used as a coating material for silicone breast implants to prevent PPC contracture and the recruitment of macrophages, neutrophils, and cellular elements responsible for contracture formation (myofibroblast). It is considering that there is a high possibility that ACMs can attenuate the inflammatory response of tissue [13].

Due to ACMs being considered scaffolds, they must accomplish the same characteristics, such as promoting the healing process, progenitor cell differentiation, and mimicking the extra-cellular environment [14]. Some researchers have described the influence of materials in stem cell differentiation where the type of material directly affects the final differentiated cell [15].

Restoring the damaged tissue requires different types of cells, appropriate ECM components, and the collaboration of cytokines. Among these cells, adipocytes, which are the primary energy storage location, have a regenerative role due to the production of different adipocytokines involved in repair and regeneration processes [16, 17].

This study aimed to investigate three natural ACMs, two porcine-derived and one bovine-derived membrane, with different physical structures, such as porosity, thickness, and stratification, both *in vitro* and *in vivo*. *In vitro* cell-biomaterial interaction was analysed through colonization capacity of adipose-derived stem cells (ASCs) in direct contact with the ACMs. Subcutaneous implantation of ACMs in mice was assessed to evaluate the tissue integration and biostimulation membrane-induced.

## Materials and methods

### *In vitro* evaluation

#### Acellular collagen membranes

In this study, three xenogeneic non-crosslinked membranes provided by DECO med S.r.l. (Venice, Italy) were evaluated:

- 0.6 mm thick porcine acellular dermis, without additional chemicals, manufactured by ADIPOMATRIX® processes (subjected to industrial secrecy) named ACM1.
- 0.6 mm thick porcine-derived dermal membrane prepared with a common enzymatic deantigenation named ACM2.
- 0.4 mm thick bovine-derived acellular pericardium membrane with a common enzymatic deantigenation named ACM3.

#### Morphological and physical properties of membranes

Scanning electron microscopy (SEM) was performed to evaluate the membranes in dry and wet conditions. Each piece was fixed for 4 h with glutaraldehyde 2% (Sigma Aldrich, Milan, Italy) in 0.1 M Phosphate Buffer Solution (PBS), postfixed in 1% osmium tetroxide (Sigma Aldrich, Milan, Italy) in PBS 0.1 M for 1 h. Then, the samples were dehydrated in a graded concentration of ethanol (Sigma Aldrich, Milan, Italy), followed by a critical point dryer (CPD 030, Balzers, Vaduz, Liechtenstein), fixed to stubs with colloidal silver and sputtered with gold by a MED 010 coater (Balzers), to be imaged with FEI XL30 scanning electron microscope (FEI Company, Eindhoven, Netherlands). The increment of mean porous size of membranes due to the embedding in culture growth media was calculated using Image-J Software using 10 SEM images taken before and after the embedding process at the same magnification. In addition, the swelling ratio of the evaluated membranes was measured using Eq. 1, where W_i_ is the initial weight of the membrane under dry conditions and W*w* the weight after submerging the membrane in PBS for 24 h at room temperature [20, 21].

1 $$S\left( \% \right) = \left( {{\raise0.7ex\hbox{${W_{w} - W_{i} }$} \!\mathord{\left/ {\vphantom {{W_{w} - W_{i} } {W_{i} }}}\right.\kern-\nulldelimiterspace} \!\lower0.7ex\hbox{${W_{i} }$}}} \right) * 100$$

The porosity percentage was evaluated according to Archimedes principle and the procedure suggested by Lou T. et al*.* [20, 21] using pure ethanol (Sigma-Aldrich, Milan, Italy) as displacement liquid. Membranes were weighed in dry (W) and subsequently soaked in 70% ethanol (Sigma-Aldrich) for 1 h in vacuum conditions. After the time passed, wet membranes were submerged in a known volume (V_1_) and weight (W_1_) of pure ethanol. The system weight (W_2_) and the displaced volume (V_2_) were measured. The porosity percentage (ε) was calculated using Eq. 2, considering pure ethanol density (ρ) at 20 °C.

2 $$\varepsilon = {{{\raise0.7ex\hbox{${\left( {W_{2} - W_{1} - W} \right)}$} \!\mathord{\left/ {\vphantom {{\left( {W_{2} - W_{1} - W} \right)} \rho }}\right.\kern-\nulldelimiterspace} \!\lower0.7ex\hbox{$\rho $}}} \mathord{\left/ {\vphantom {{{\raise0.7ex\hbox{${\left( {W_{2} - W_{1} - W} \right)}$} \!\mathord{\left/ {\vphantom {{\left( {W_{2} - W_{1} - W} \right)} \rho }}\right.\kern-\nulldelimiterspace} \!\lower0.7ex\hbox{$\rho $}}} {\left( {V_{2} - V_{1} } \right)}}} \right. \kern-\nulldelimiterspace} {\left( {V_{2} - V_{1} } \right)}}$$

#### Isolation and seeding of adipose-derived stem cells

ASCs were isolated from human lipoaspirate of healthy donors (women of ages between 35 and 45 years) after informed consent, following the protocol described by Peroni et al. [22] and Busato et al*.* [23], using an enzymatic method [18, 19]. The lipoaspirate samples were incubated in 1 mg/mL of Collagenase type I (GIBCO Life Technology, Monza, Italy) dissolved in Hank’s Balanced Salt Solution (HBSS, GIBCO Life Technology) with 2% of Bovine Serum Albumin (BSA, GIBCO Life Technology). Complete growth medium (Dulbecco’s Modified Eagle’s Medium (DMEM), Sigma-Aldrich) supplemented with 10% of fetal bovine serum (FBS, GIBCO Life Technology), 1% of 1:1 penicillin/streptomycin (P/S, GIBCO Life Technology) solution, and 0.6% of amphotericin B (GIBCO Life Technologies) was added to neutralise the enzymatic action. The extracted cells were incubated in a humidified atmosphere with 5% CO_2_ at 37 °C in a 25 cm^2^ flask with complete growth medium. The cells were detached after reaching between 70 and 80% confluence by incubation with 0.25% trypsin (GIBCO Life Technology) at 37 °C for 5 min, centrifuged at 3000 rpm for 7 min, and the cell pellet was re-plated in a 25 cm^2^ flask. The cells were cultured until passage four (P4), following the procedure mentioned above.

#### ASCs colonization capacity and *in vitro* biostimulation

For each ACM, two 6-well plates were prepared for *in vitro* test, consisting of the incubation of a 1 cm^2^ of the membrane with P4 cultured ASCs, for 7 and 14 days. For this aim, a glass was positioned at the bottom of the wells and simultaneously 1 × 10^4^ cells were seeded in each well, covered with 2 ml of complete growth medium and incubated at 37 °C, 5% of CO_2_ for 24 h. After incubation, 1 cm^2^ of each ACM was positioned in the wells (with both layers facing the bottom of the well) and incubated for 24 h with complete growth medium (DMEM supplemented with 10% FBS, 1% P/S and 0.6% amphotericin B). After 24 h, ASCs were homogeneously seeded above and over the membranes. All the conditions were performed in triplicates. For all the wells, complete growth medium was replaced every 72 h.

After 7 and 14 days, the medium was discarded and, both membranes and cells were processed for SEM (with the protocol reported above) and oil red O staining, respectively. Briefly, once the membranes were removed from the 6-well plate, the coverslips with adherent ASC were washed with PBS 0.1 M pH 7.4 and fixed for 30 min with 4% formalin (Bio-Optica, Milan, Italy) in PBS 0.05 M. The adherent cells were washed with PBS 0.1 M three times and stained with oil red O ready-to-use solution (Bio-Optica) for 30 min at room temperature. Samples were washed with PBS and stained with Mayer’s hematoxylin ready-to-use solution (Bio-Optica) for 2 min, at room temperature, and washed with tap water. The coverslips were mounted on a microscopy glass with a Mount Quick aqueous solution (Bio-Optica). Once the microscopy glasses were dried under the cabinet, samples were observed in light microscopy using an Olympus BX-51 microscope (Olympus, Tokyo, Japan) equipped with a DKY-F58 CCD JVC digital camera (Yokohama, Japan) with magnification 20X.

ASCs cultured with complete growth medium (without ACMs) were used as a negative control. Instead, ASCs cultured with specific adipogenic media (Sigma-Aldrich) were used as a positive control.

### *In vivo* evaluation

#### Membranes subcutaneous implant in mice

For *in vivo* study were used n = 30 Balb/c female mouse strain of 10-weeks old purchased from Envigo (Envigo, Milan, Italy) implanted in the left flank, 10 mice for each evaluated membrane. Animals were housed in a controlled environment, as indicated by the Interdepartmental Center for Animal Study and Research of Verona University (CIRSAL), with free access to food and water. The protocol of membrane implant was approved by CIRSAL and by the Italian Ministry of Health (protocol number 56DC9.38). For the subcutaneous implant, animals were anaesthetized using a face mask by inhalation of 2% isoflurane for 5 min and left at 1% for the duration of surgery. Moreover, animals were positioned on a heated bed to maintain a stable temperature during surgical procedures.

A subcutaneous pocket was made at the dorsal region, and 1 cm^2^ of each membrane was implanted above the muscular fascia followed by a suture using a non-absorbable silk 3/8 13 mm 4.0 suture-thread. At the end of the surgery, animals were housed following the CIRSAL guidelines. Three mice for each group were sacrificed 7 days after surgery, three after 14 days, and the remaining mice were sacrificed at 30 days.

In addition, silicone prostheses were implanted in a group of n = 3 animals (positive control) using the previously described protocol, and the animals were sacrificed 30 days after surgery.

#### Magnetic resonance imaging (MRI) acquisition

MRI was performed at different time points (7, 14, and 30 days after subcutaneous implantation) to evaluate the membranes and silicone prosthesis localisation and integration. Magnetic resonance images were acquired with a Bruker system operating at 7 T (Bruker Biospin, Ettlingen, Germany). T_2_ weighted 3D RARE (Rapid Acquisition Refocused Echo) sequence was performed with the following parameters: echo time (TE) = 4 ms, repetition time (TR) = 1200 ms, field of view (FOV) = 25 × 25 × 30 mm, number of averages = 16, flip angle = 180 degrees, slice thickness = 0.350 mm, and matrix size (MTX) = 256 × 128 × 32 pixels. During the procedure, mice were anaesthetized by inhaling a mixture of O_2_ and air containing 1–1.5% of isoflurane, placed in a prone position in the heated animal bed in a 3.5 cm diameter bird-cage coil. The acquisition parameters were maintained during the time of observation for all the animals. Moreover, the volume of each subcutaneous implant was determined with the MRI acquisition to quantify the reabsorption degree at different time points. The area occupied by the subcutaneous implant was manually selected, drawing a Region of Interest (ROI) in every slice, and the volume was calculated with Eq. 3.

3 $$V=\left({\sum }_{i=1}^{n}\rho {u}_{i}\right)*image\,resolution$$

where ρu_i_ represents the number of pixels manually selected.

The percentage of membrane reabsorption was calculated at each time point using Eq. 4:

4 $$Reabsorption = \left( {{\raise0.7ex\hbox{${\left( {V_{t} - V_{0} } \right)}$} \!\mathord{\left/ {\vphantom {{\left( {V_{t} - V_{0} } \right)} {V_{0} }}}\right.\kern-\nulldelimiterspace} \!\lower0.7ex\hbox{${V_{0} }$}}} \right)*100$$

where V_t_ is the volume at a time *t* and V_0_ is the initial volume.

#### *Ex vivo *evaluations

After MRI acquisition at 14 and 30 days from subcutaneous implantation, mice were sacrificed, and ACMs with surrounding tissue was excised. The explanted samples were fixed in 10% formalin for 24 h, paraffin-embedded and cut with a microtome to obtain sections of 7 µm. For the histological evaluation, the slices were deparaffinised and stained with Mayer’s hematoxylin and Eosin (H&E, Bio-Optica) and Mallory’s trichrome (MT, Bio-Optica). The same histological stainings were performed for the excised tissue exposed to the silicone prostheses after 30 days of implantation.

In addition, the samples were processed with collagen type I (Coll. I) and vascular endothelial growth factor (VEGF) for immunohistochemical analysis to evaluate the tissue-ACMs integration. The slides were deparaffinized in xylene for 20 min, rehydrated and incubated with Sodium citrate buffer (10 mM sodium citrate and 0.05% of Tween 20, pH 6.0) for 20 min at 95–100 °C to promote the antigen retrieval procedure. After slow cooling, the slices were incubated in hydrogen peroxide (3% in methanol) (Sigma-Aldrich) for 30 min to cease the endogenous peroxidase activity. The slices were washed in phosphate-buffered saline (pH 7.4) and incubated with blocking solution (10 g/L BSA, 3 mL/L Triton X-100 and 10 mL/L normal goat serum) for 30 min. The samples were incubated with the following primary antibodies overnight at 4 °C: rabbit anti-collagen type I, dilution 1:500 (GTX41286; GeneTex, Irvine, CA, USA); mouse anti-VEGF, dilution 1:100 (GTX83426; GeneTex). After a short washing in phosphate-buffered saline (pH 7.4), the slices were covered with biotinylated secondary antibody (anti-rabbit antibody diluted 1:400 for collagen type I; anti-mouse antibody diluted 1:400 for VEGF) for 1 h at room temperature. The samples were then incubated with avidin–biotin complex (VECTASTAIN Elite ABC-HRP Kit, Vector Laboratories, Burlingame, CA, USA) for 45 min at room temperature, and the immunoreaction was identified adding 3,3’-diaminobenzidine tetrahydrochloride (Dako, Santa Clara, CA, USA) for 5 to 10 min. Finally, the sections were re-dehydrated and mounted with Entellan (Merck, Kenilworth, NJ, USA). The glasses were examined by light microscopy using an Olympus BX-51 microscope, equipped with a DKY-F58 CCD JVC digital camera.

In order to characterize the adipogenic biostimulation, the newly formed adipose tissue was analysed at 30 days for mouse anti-collagen type III (Coll. III), dilution 1:1000 (GTX26310; GeneTex) and VEGF using the same protocol previously described.

## Results

### *In vitro* evaluation

#### Morphological and physical characterization

Structural and physical properties of the evaluated membranes were measured in order to analyse the morphological characteristics and the porosity in dry and wet conditions in culture growth media.

**Fig. 1:**
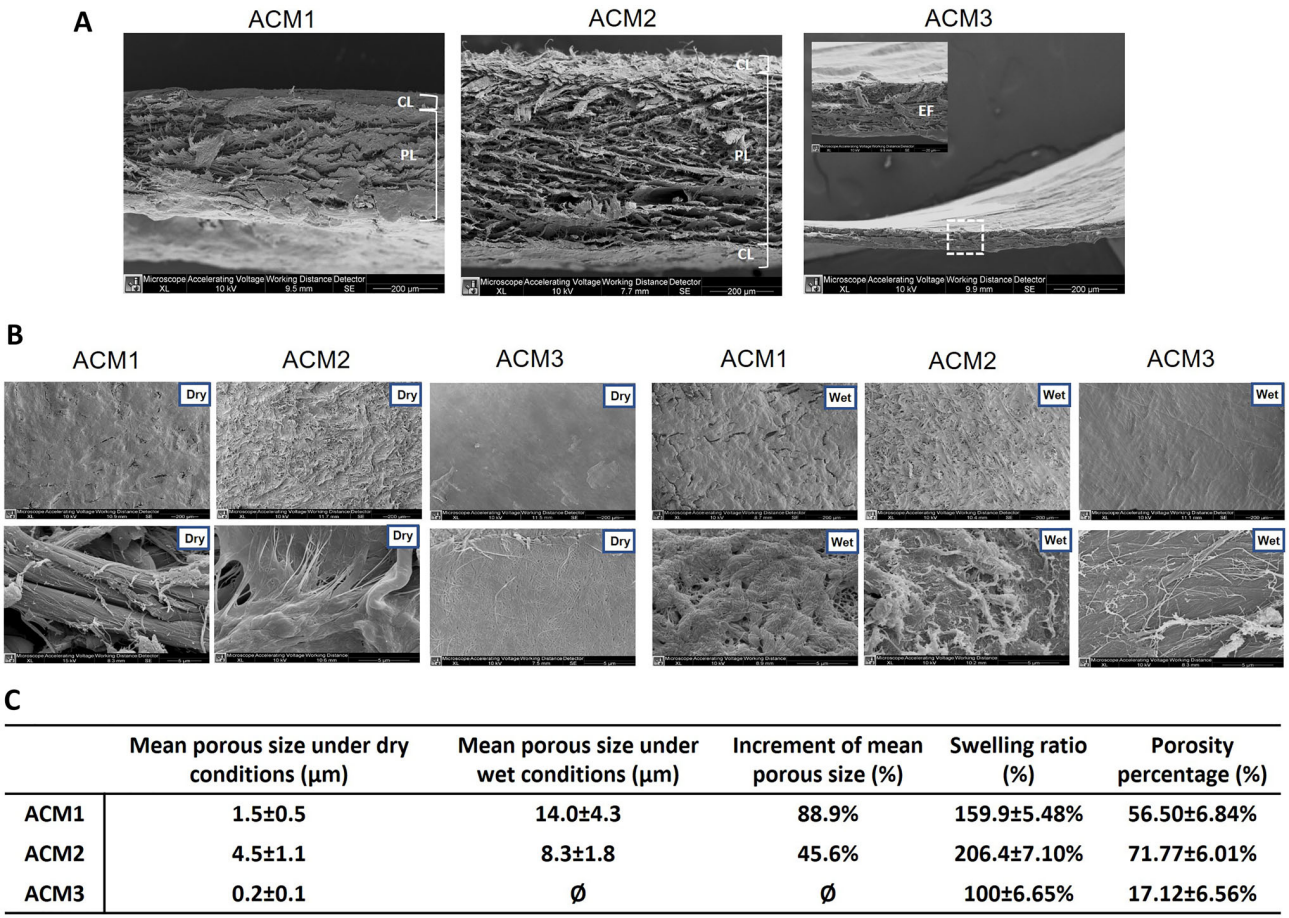
Morphological and physical characterization of the ACMs. **A** SEM of ACMs in cross-view. The layered structure is identifiable for ACM1 and ACM2 (white bars indicates the different layers, Scale bar 200 µm), while ACM3 is composed of a single layer (Scale bar 200 µm) and are recognizable the elastic fibres (white square indicates the area of higher magnification, Scale bar 20 µm). **B** SEM images of ACMs at top view at two magnifications in dry and wet conditions (Scale bar: upper 200 µm, bottom 5 µm) show the surface porosity created by the crossed collagen fibres. **C** The table summarizes the physical properties of ACMs (data reported as mean ± standard deviation). *CL*: Compact layer; *PL*: Porous layer; *EF*: Elastic fibres

Figure 1A shows the SEM images at cross-view in dry conditions of the ACMs. The stratification is noticeable due to the different pressing grades of ACM1 and ACM2, which present two and three layers, respectively. While ACM3 results composed by a single stratum where the elastic fibres (EF) are clearly identifiable, as indicated in the higher magnification reported in Fig. 1A. ACM1 presents a two-layer structure, one is highly porous (porous layer: PL), while the other appears more compact (compact layer: CL). SEM images reveal that ACM2 is composed of three layers; both external layers are thinner and more compact (CL), while the middle one results porous (PL).

Figure 1B presents the top view of the membranes in dry and wet conditions at two different magnifications. ACM1 exhibits a porous surface composed of crossed collagen fibres forming an irregular porosity through 3D structure with a mean porous size near 2 µm as reported in Fig. 1C. After the embedding procedure in culture growth media, it can be seen that the lamellar organization is more noticeable, and the size of the porous is increased. In the case of ACM2, the surface displays a porous configuration when analysed dry with a mean porous size ranging between 4 and 5 µm (Fig. 1C). After embedding in culture growth media, the lamellar structure appears non-homogenous, and the porous size of the membrane surface increases (from 4.5 ± 1.1 to 8.3 ± 1.8 µm), as reported in Fig. 1C.

Likewise, ACM3 was analysed before and after embedding in culture growth media. Under dry conditions, the membrane surface is highly dense and smooth, composed of a compact fibre structure that creates porous (0.2 ± 0.1 µm) as reported in Fig. 1C. After the embedding process, the membrane is characterised by a more irregular surface and the porous results closed due to the liquid uptake.

The table in Fig. 1C reports the swelling ratio and the porosity percentage. It is appreciable that liquid absorption capacity influences the porosity and, therefore, the increment of porous size. For ACM1, the increment of mean porous size is higher than the reported for the other two evaluated membranes (88.9%) with a porosity percentage of (56.50 ± 6.84%). On the contrary, for ACM2, the swelling ratio is higher, which probably increases the size of the fibres that might affect the increase of the porous size (45.6%), and in addition, its porosity percentage is increased (71.77 ± 6.01%). The same effect is reported for ACM3, which loses its porosity due to liquid uptake (swelling ratio of 100 ± 6.65%), affecting its porosity percentage (17.22 ± 6.56%).

#### ASCs colonization capacity

**Fig. 2:**
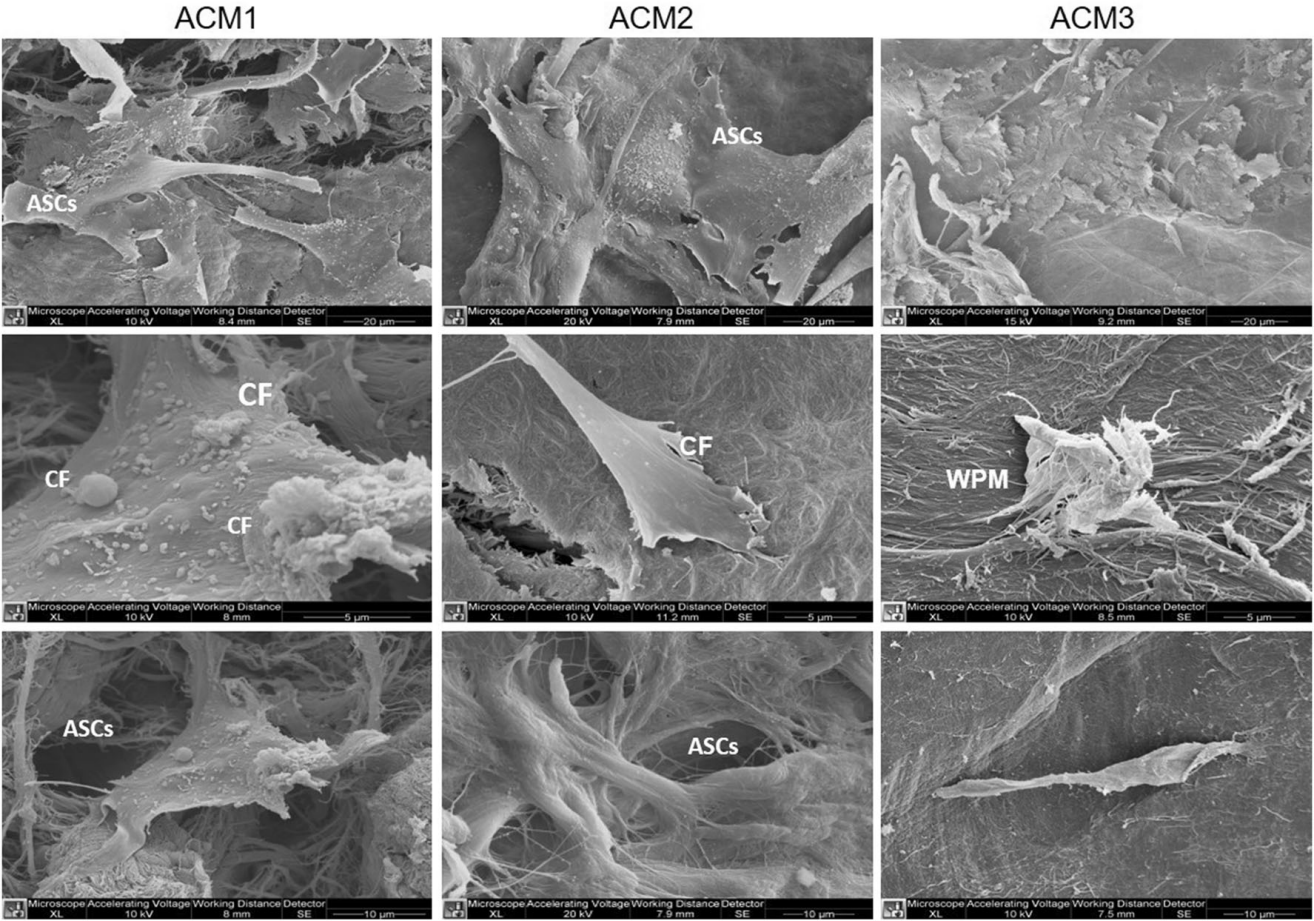
SEM analysis of ASCs colonization capacity over ACMs at different magnifications. Some cytoplasmic flaps are appreciable on cells cultured with ACM1 and ACM2, while cells in contact with ACM3 present a wrinkled plasmatic membrane. (Scale bar: upper 20 µm, middle 5 µm, bottom 10 µm) (*ASCs*: Adipose-derived stem cells on ACMs surface and spreading through their porous, *CF*: Cytoplasmatic flaps on ASCs Surface, *WPM*: wrinkled plasmatic membrane)

To evaluate the colonization capacity of ASCs on ACMs surfaces, the cells were cultured over the membranes for 7 days and subsequently were studied with SEM images. Figure 2 shows on the upper panel the membranes surface colonized by cultured cells. It is appreciable that for ACM1 and ACM2, the surface is covered with ASCs with elongated filopodia, while for ACM3 cells are hardly detectable. Seeing the middle panel, cells in contact with ACM1 reveal the presence of numerous cytoplasmic flaps (CF) over the surface, which indicates strict adherence with the membrane. The CF detectable on the ASCs surface in contact with ACM2 appears scarce. The cells in contact with ACM3 present a non-conventional shape with a wrinkled plasmatic membrane (WPM). Furthermore, the adhesion between cells and the membrane is reduced.

The last panel shows the cells growing and spreading through the porous of ACMs colonizing the 3D structure. Those cells seeded over ACM1 and ACM2 generate connections between them; while, in the case of ACM3, the cells are not adherent to the surface and are not characterised by the presence of filipodia.

#### *In vitro* biostimulation

**Fig. 3:**
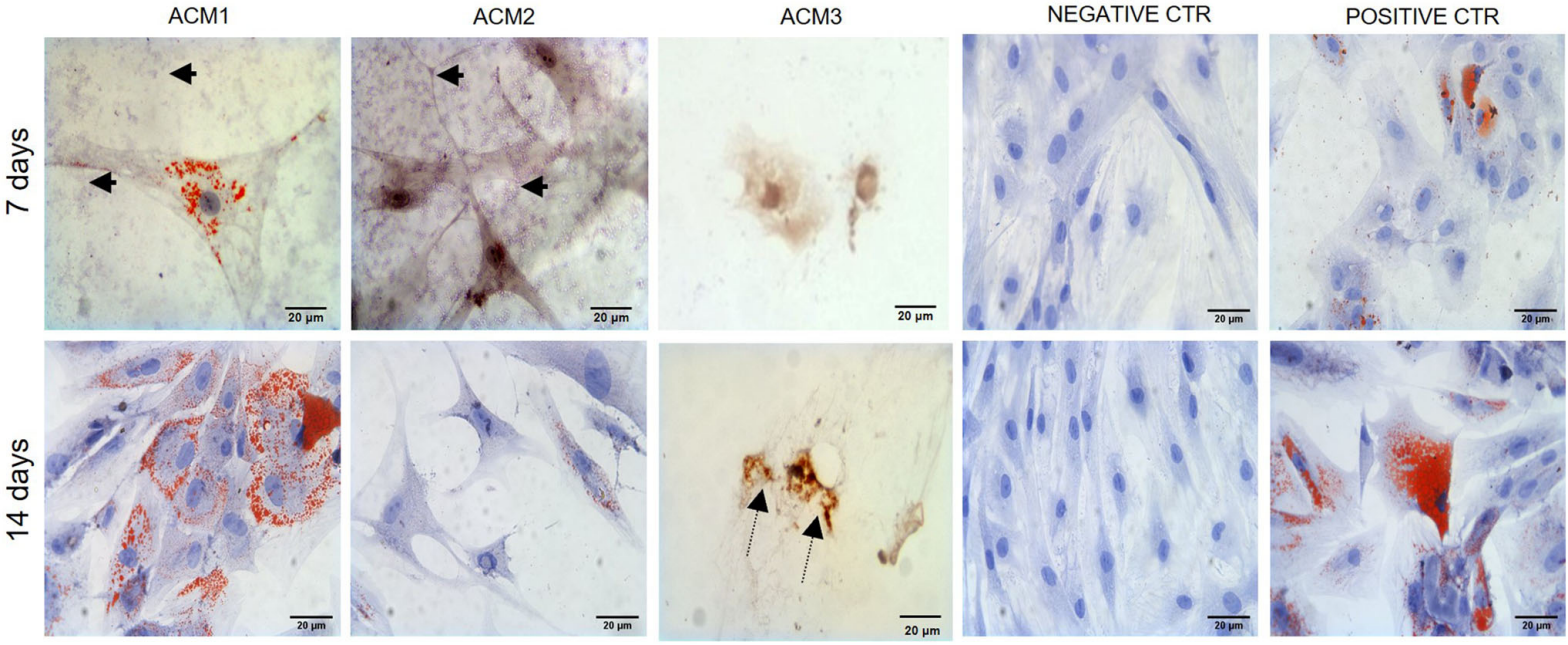
In vitro biostimulation. ASCs after 7 and 14 days of contact with the evaluated ACMs were compared with ASCs cultured growth medium (negative control) and adipose differentiative medium (positive control). (Scale bar 20 µm) (Arrows indicate membrane debris, dotted arrows indicate vacuolization)

ASCs attached to the bottom of wells in which the ACMs were placed for 7 and 14 days were stained with Oil-Red-Oil solution and compared with positive and negative controls at the same time points. Figure 3 shows the images of the stained cells at the same magnification. On day 7, ASCs in contact with ACM1 started to internalise lipid droplets seen as red spots on the cytoplasm. The amount and size of the lipid droplets indicate an early stage of differentiation compared with the positive control. Additionally, small amounts of membrane debris around the cells can be seen in the culture medium (Fig. 3, black arrows). On the other hand, ASCs in contact with ACM2 do not present lipid droplets on their cytoplasm, appearing with the typical morphology of stem cells as compared with the negative control. Moreover, a high quantity of membrane debris is detectably dispersed among the cells, probably due to the elevated degradation rate of the membrane. The cells cultured with ACM3 are reduced in number and appear stressed with a suffering morphology.

After 14 days of study, ASCs exposed to ACM1 show their capacity to duplicate actively, reaching the confluence and with a visible increment of lipid droplets, indicating the progression in adipogenic differentiation similar to the reported for positive control. ASCs growing in the presence of ACM2 starts to accumulate lipid droplets in the cytoplasm, indicating a slow ASCs differentiation into adipocytes compared to the positive control and cells in contact with ACM1. Finally, for ACM3, the behaviour found on the 7th day continues in time, showing that the membrane affects both the growth, the shape, and the vacuolization (Fig. 3, dotted arrows) of ASCs. No adipose differentiation is visible.

### *In vivo *and *ex vivo* evaluation

#### Tissue integration

**Fig. 4:**
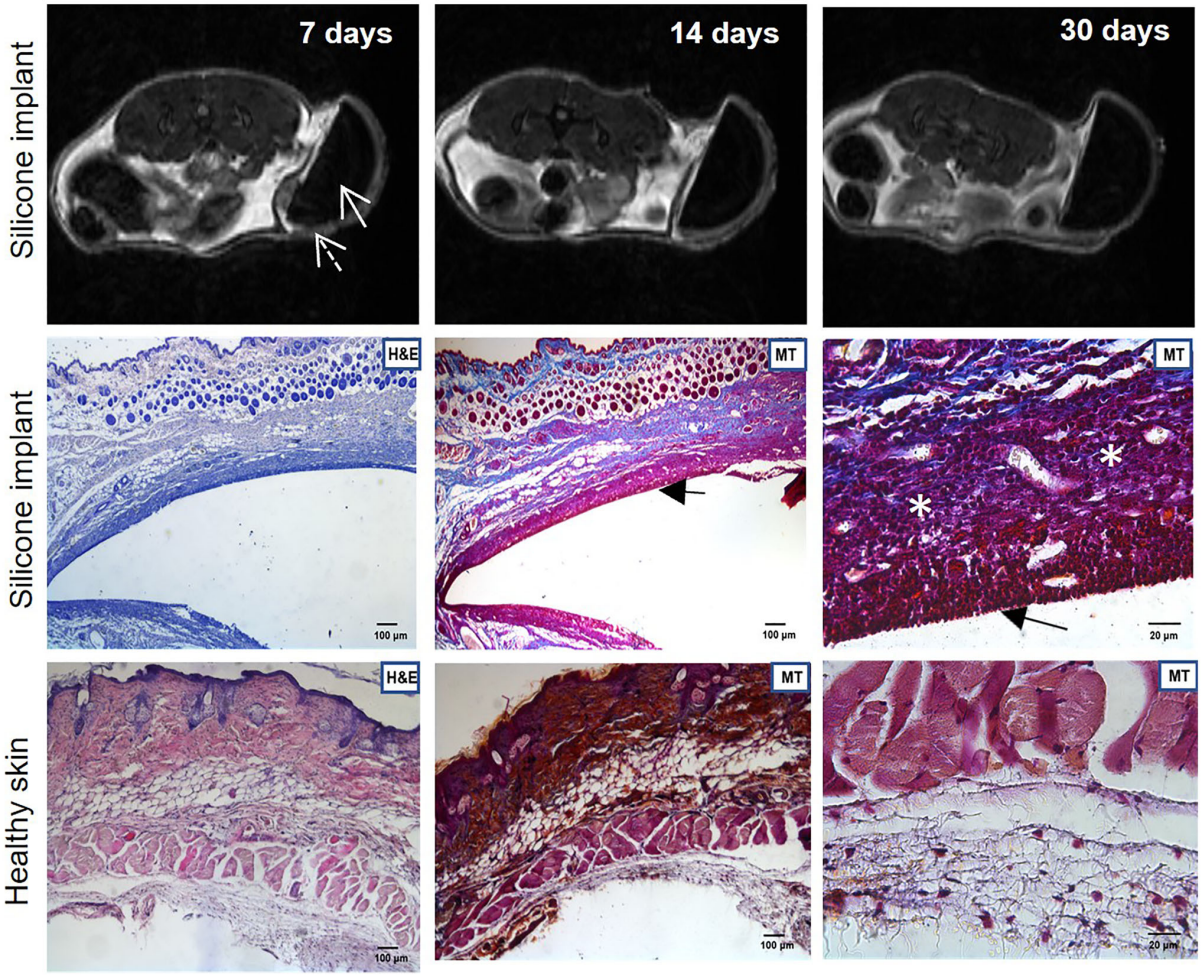
Elastosis formation after silicone prosthesis implantation. Magnetic Resonance images of control group are shown on the upper line after 7, 14 and 30 of follow-up. Histology images on the middle and bottom panels show H&E (Scale bar 100 µm) and MT (Scale bar 100 µm and 20 µm) staining of negative control group (uncovered silicone prosthesis) compared with positive control (healthy skin). (Arrow: silicone prosthesis; dotted arrow: fluid accumulation: black arrow: elastosis, White asterisks: Inflammatory reaction, *H&E*: Hematoxylin/Eosin staining; *MT*: Mallory’s Trichrome)

In order to evaluate how silicone prosthesis affects the tissue, MR images and histological analysis were performed on mice subcutaneously implanted with silicone prosthesis. The MRI was performed for 7, 14 and 30 days after the silicone implantation. The images clearly identify the area of the silicone graft (Fig. 4) surrounded by an area of fluid accumulation (Fig. 4, dotted arrow) that appears stable over time. The ex vivo findings display an altered tissue morphology in the area of silicone prosthesis insertion (Fig. 4, H&E) compared to the positive control (Fig. 4, healthy skin H&E). Analysing the tissue with MT is possible to identify an accumulation of elastic fibres (Fig. 4, MT black arrows) surrounded by an inflammatory infiltration (Fig. 4, MT white asterisks) on the site of contact with the silicone implant. MT staining of healthy skin shows the physiological and non-altered subcutaneous structure.

**Fig. 5:**
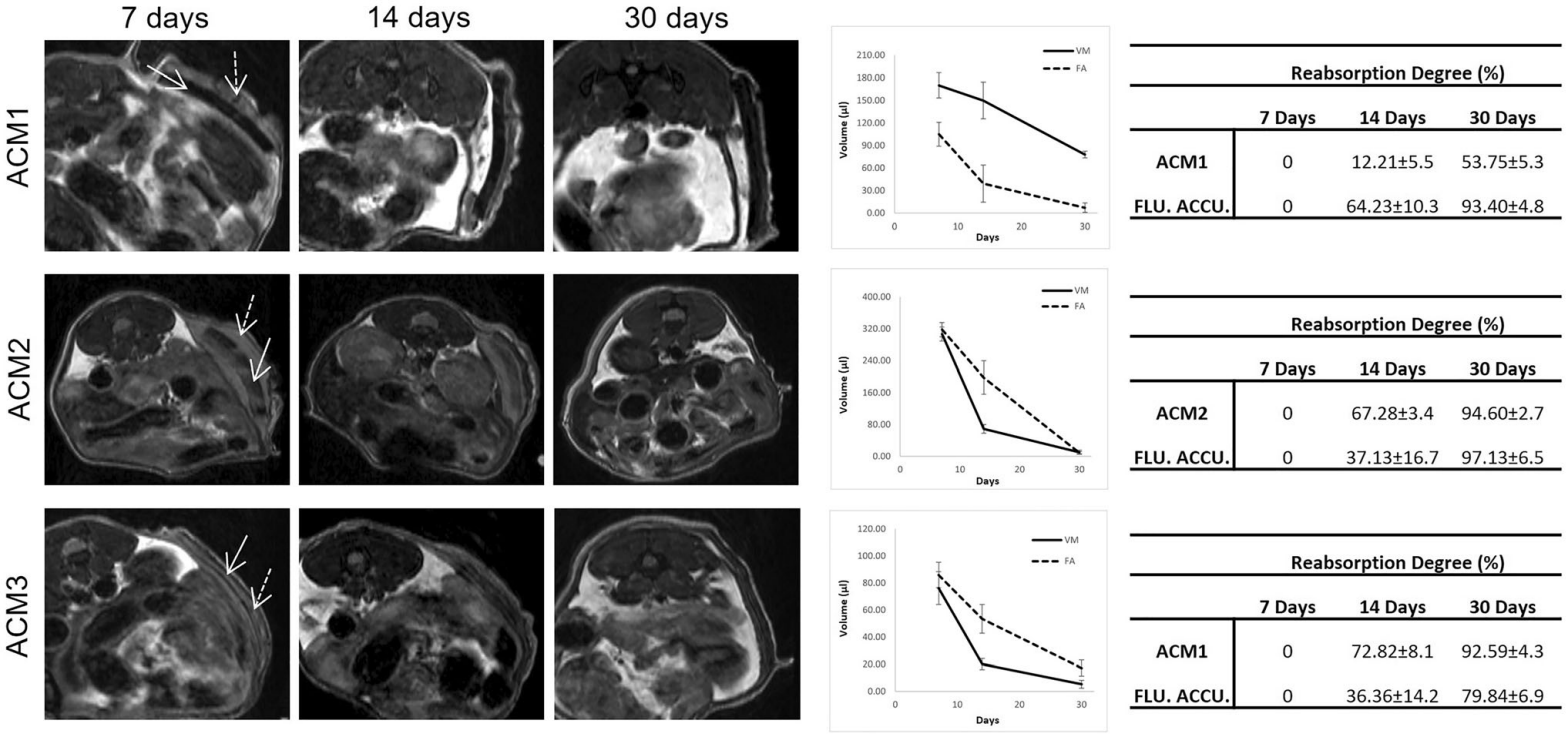
Evaluation of *in vivo* tissue integration. Magnetic resonance images performed after 7, 14, and 30 days of implantation of the evaluated ACMs allowed the calculation of the membrane’s reabsorption degree and the surrounding fluid accumulation. (Arrow: ACMs, dotted arrow: fluid accumulation)

The in vivo tissue integration induced by the evaluated membranes was assessed after the subcutaneous implantation in mice of the ACMs. Figure 5 shows the MRI acquisitions after 7, 14 and 30 days from the surgery. On day 7th of study, the membranes are completely visible (Fig. 5, arrows), and a fluid accumulation (Fig. 5, dotted arrows) is observed in the subcutaneous region adjacent to all the evaluated grafts. After 14 days of study, the membranes reabsorption degree was calculated (Fig. 5, reabsorption degree tables). Images reveal minimal reabsorption of ACM1 (12.21 ± 5.5), while the reabsorption rate of ACM2 and ACM3 results faster (67.28 ± 3.4 and 72.82 ± 8.1, respectively). On the contrary, the fluid accumulation bordering the ACM1 seems to be reabsorbed faster than ACM2 and ACM3 (64.23 ± 10.30, 37.13 ± 16.70 and 36.36 ± 14.20, for ACM1, ACM2 and ACM3, respectively).

On the 30th day of follow-up, the fluid accumulation is not already visible in the surroundings of ACM1 with a reabsorption degree of 93.40 ± 4.8%, while only 53.75 ± 5.3% of the membrane was reabsorbed. At the same time point, both ACM2 and the fluid accumulation around the membrane were almost entirely reabsorbed with a reabsorption degree of 94.60 ± 2.7% and 97.13 ± 6.5%, respectively. In the case of ACM3, while the fluid accumulation is still present (reabsorption degree of 79.84 ± 6.9%), the membrane appears highly reabsorbed (reabsorption degree of 92.59 ± 4.3%), and the remains appear adherent to the muscular tissue of the animal.

**Fig. 6:**
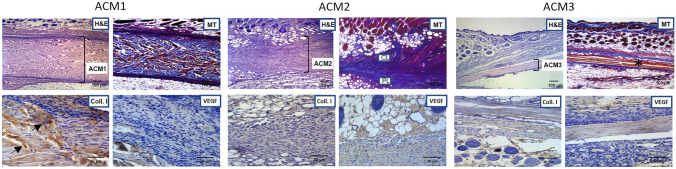
*Ex vivo* tissue integration after 14 days of study. The excised samples (ACMs with surrounding tissue) were stained with H&E and MT showed in upper panel (Scale bar 100 µm). Additionally, immunohistochemical analyses were performed with Coll. I and VEGF antibodies in bottom panel (Scale bar 30 µm). Asterisk indicates loss of fibre density; arrows indicate Coll. I positivity. *CL* Compact layer, *PL* Porous layer, *H&E*: Hematoxylin/Eosin staining, *MT*: Mallory’s trichrome, *Coll. I*: Collagen type I staining, *VEGF*: Vascular Endothelial growth factor staining

After 14 and 30 days from implantation, the ex vivo examinations were performed to analyse the morphological structure modifications of ACMs (H&E and MT staining) and tissue integration and colonization (Coll. I and VEGF) as shown in Fig. 6.

After 14 days of surgery, histological examinations confirm the MRI results, and all the ACMs are still distinguishable. ACM1 appears well preserved, and the three-layer results are non-altered, as shown in Fig. 6 (H&E and MT). As confirmed with the immunohistochemical analysis. Mallory’s staining revealed that the membrane was colonised by resident cells and collagen fibre (Fig. 6, Coll. I). Indeed, the positive result for the collagen type I antibody suggests an early integration between the autologous connective tissue and the membrane (Fig. 6, Coll. I, arrows). No positive expression of VEGF was found.

Regarding ACM2, the membrane is well visible, and it is possible to recognise the layered structure (Fig. 6, H&E and MT): CL has been positioned immediately after the reticular dermis, and PL is placed on the opposite side (Fig. 6, MT). The immunohistochemical analysis shows no migration of native collagen fibres nor new vessels formation in the membrane (Fig. 6, Coll. I and VEGF). However, the membrane results highly infiltrated by cells, probably due to an inflammatory reaction. For ACM3, the membrane appeared dense and compact (Fig. 6, H&E and MT) except in the central portion in which the fluid accumulation has caused the loss of fibres density (Fig. 6, MT, asterisk). Moreover, thanks to MT staining, the architecture of ACM3 is clearly visible: the elastic fibres that typically compose the pericardium are detectable as the red fibres in Fig. 6. No evidence of cells colonisation (Fig. 6, H&E), resident collagen fibres infiltration (Fig. 6, Coll. I), and neovascularization (Fig. 6, VEGF) are detectable.

**Fig. 7:**
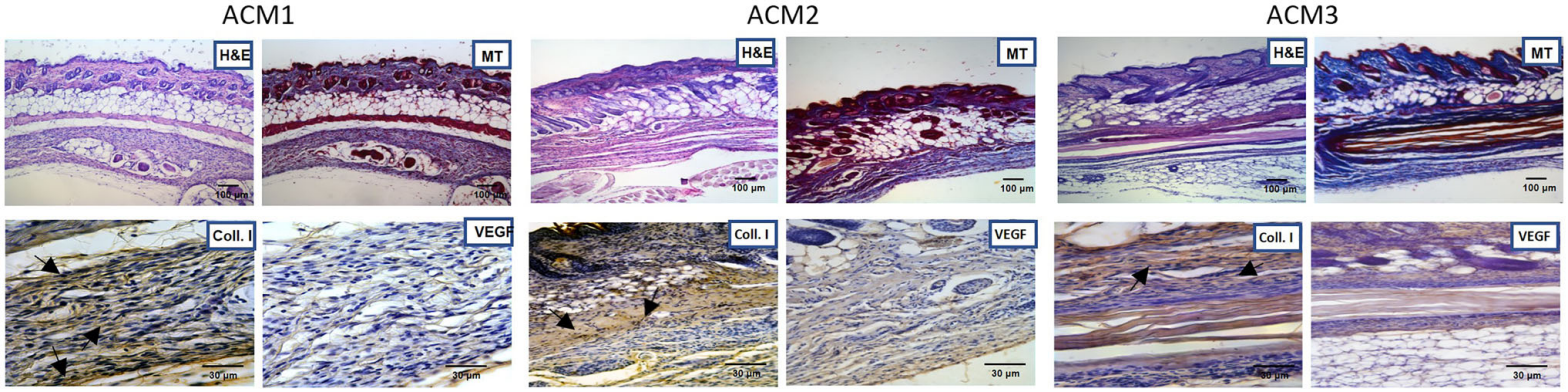
*Ex vivo* tissue integration after 30 days of study. The excised samples (ACMs with surrounding tissue) were stained with H&E and MT are shown in upper panel (Scale bar 100 µm). Additionally, immunohistochemical analyses were performed with Coll. I and VEGF antibodies in bottom panel (Scale bar 30 µm). Arrows identify positive responses for Coll. I. H&E: Hematoxylin/Eosin staining, *MT*: Mallory’s Trichrome, *Coll. I*: Collagen type I staining, *VEGF*: Vascular Endothelial Growth Factor staining

At 30 days after the surgery, the ACM1 shows a reduced stratification and results partially reabsorbed, in accordance with MRI findings (Fig. 7). Moreover, the membrane appears compact with a well-structured collagen fibre (Fig. 7, MT). Comparing the results with those at 14 days, ACM1 seems homogenously colonized by resident cells and collagen fibres (Fig. 7, Coll. I, arrows) but with no new vessel formation identified (Fig. 7, VEGF).

In the case of ACM2, the dermis of the mice shows a physiological organisation while the membrane is hardly detectable (Fig. 7, H&E and MT), probably due to the high degree of reabsorption as reported by MRI investigation. Moreover, an organically structured layer of resident collagen fibres (Fig. 7, Coll. I, arrows) is detectable near the ACM2 implantation site, suggesting that the dermal compartment is fully restored, but the membrane results less colonized by collagen fibres than ACM1. Immunohistochemical analysis for neo-vascularization reveals no positive response for ACM2 (Fig. 7, VEGF).

Finally, histological analysis of ACM3 confirms that the membrane is completely adherent at the muscular tissue and an unfolded, not compact, and disorganized structure is clearly identifiable (Fig. 7, H&E and MT), justifying the high reabsorption degree obtained with MRI images. Immunohistochemical analysis reveals a partial integration between the membrane and the surrounding tissue. Native collagen fibres (Fig. 7, Coll. I, arrows) and resident cells start to migrate through the ACM3. VEGF analysis reveals no signal of neo-angiogenesis surrounding the ACM3 after 30 days.

#### *In vivo* adipogenic biostimulation

**Fig. 8:**
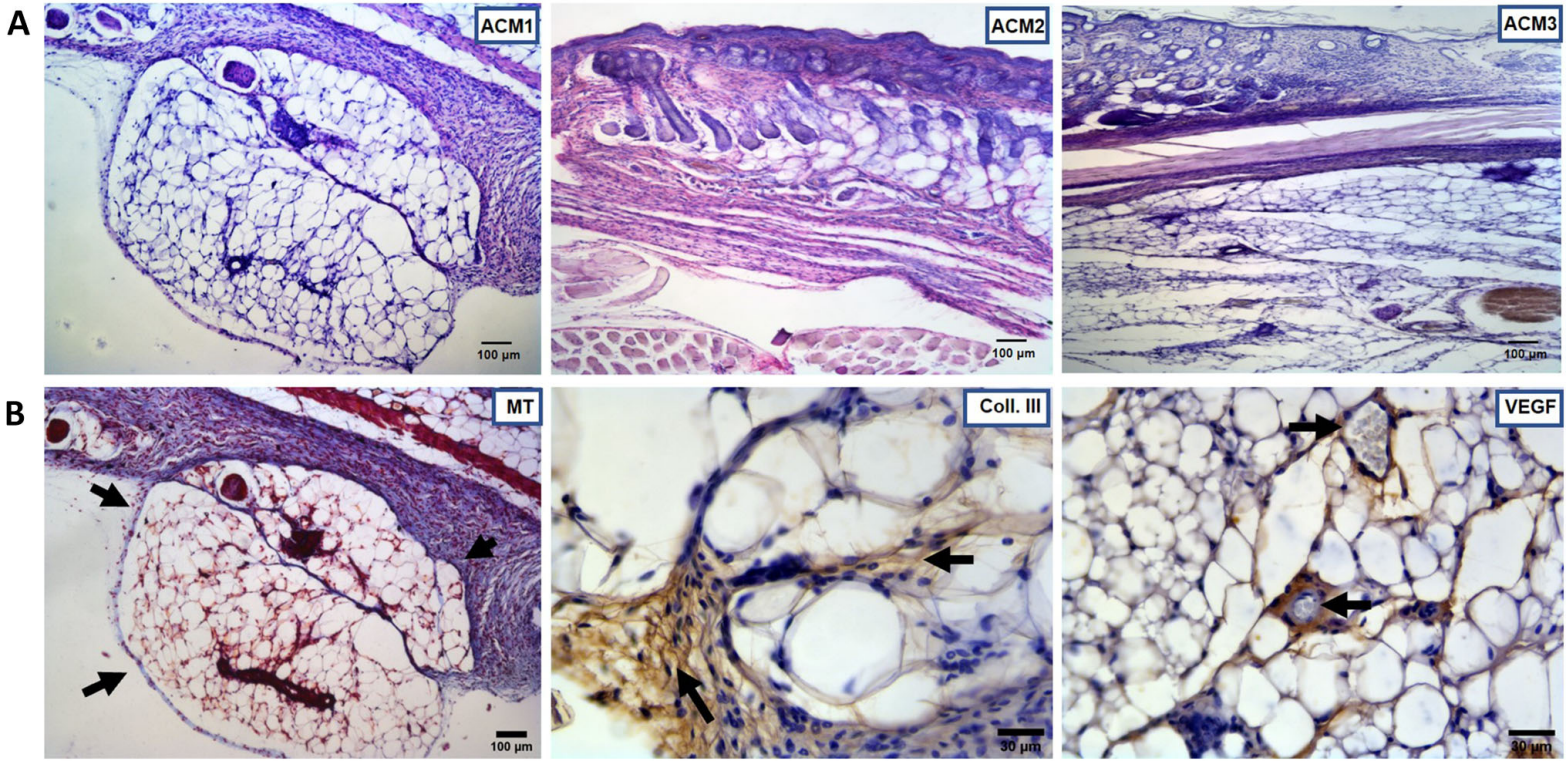
Induced adipose biostimulation study. **A** shows H&E of the evaluated membranes (ACM1, ACM2 and ACM3) revealing an adipogenic formation inside ACM1. **B** MT staining of newly formed adipose tissue inside ACM1 (arrows delimited the membrane, scale bar 100 µm) and immunohistochemical analysis of newly formed adipose tissue for Coll. III and VEGF antibodies (scale bar 30 µm). *H&E*: Hematoxylin/Eosin staining, *MT*: Mallory’s trichrome, *Coll. III*: Collagen type III staining, *VEGF*: Vascular endothelial growth factor staining

Analysing the ACMs-induced biostimulation after 30 days of study, histological examination reveals an adipogenic induction thanks to ACM1, but no signs were found on ACM2 and ACM3 (Fig. 8). Mallory’s trichrome shows a well-organised adipose tissue deposition inside the membrane (Fig. 8B). Mature adipocytes resulted connected through a thin extra-cellular matrix of newly formed collagen fibre (Fig. 8B), and immunohistochemical analysis for VEGF detects the formation of new vessels distributed in the adipose tissue (Fig. 8B).

## Discussion

The number of women undergoing breast reconstruction surgery after mastectomy through silicone implant-based procedure is steadily increasing; however, silicone or polyurethane prostheses are associated with foreign body response which results in the PPC formation [24, 25]. An unsolved issue is the elevated frequency of CC, in particular, related to breast irradiation. The CC is an inflammatory reaction characterised by fibrosis surrounding the implant due to the action of excessive collagen production, which provokes painful deformity [26].

To date, the standard treatment for CC is surgical remotion which is a highly invasive procedure. For this reason, the development of non-invasive approaches to prevent CC is necessary. Many positive reports are emerging about using biological materials to improve alloplastic breast reconstruction [25]. In this study, ACMs different in morphology and physical structure have been evaluated *in vitro* and after subcutaneous implant in mice. Firstly, ACMs were incubated with ASCs to assess the interaction between the acellular scaffold and mesenchymal stem cells, representing one of the cellular elements involved in regeneration and repair pathways [27]. According to the literature, the stimulation and differentiation of ASCs increase wound repair and regeneration ability by intensifying collagen secretion and deposition [9, 14]. Recent studies have demonstrated a link between adipocytes and wound healing, given that adipocytes also act as key regulators of skin health [28]. Collected data showed that *in vitro* colonisation capacity and adipogenic differentiation potential of ASCs are membrane dependent. Our results show that the ASCs in contact with the three evaluated ACMs have different behaviour. In particular, the high swelling ratio (more than 200%) of ACM2 improves the surface ASCs colonisation, but the non-homogenous lamellar structure and the pores mean diameter (about 8 µm) after the embedding procedure reduce the ability of ASCs to move across the 3D structure. Additionally, the cells in contact with ACM2 exhibit few cytoplasmic flaps indicating a poor activation capability. In the case of ACM3, the direct contact of ASCs with this material leads to cell stress. Indeed, SEM and optical microscopy evaluation showed a non-conventional shape of ASCs with a wrinkled and damaged cytoplasmic membrane. On the contrary, ACM1 presented an appropriate swelling ratio (about 160%) that increases the mean porous diameter (88. 9%), allowing the complete colonisation of cells through the 3D structure. The numerous cytoplasmic flaps found on the ASCs surface reveal that ACM1 is able to stimulate and activates the cells. Indeed, Dasgupta et al. reports that an increased number of cytoplasmatic processess positively affects the attachment and the spreading of cells on biomaterial surfaces [29]. Moreover, ASCs resulted stimulated by ACM1 improving their differentiation capacity into adipose-like cells.

The ACMs physical properties also influenced the degree of integration with the resident tissue and biostimulation (such as adipogenic stimulation, angiogenesis, or new collagen fibres deposition) *in vivo*. The control group implanted with the silicone prosthesis showed the formation of a high elastosis in the surroundings of the implant. On the contrary, ACMs presented a different tissue response. This suggests that using biological materials as implant beds avoiding direct contact between the silicone prosthesis and the resident tissue could favour a physiological integration [30]. Schmitz et al. [25] reported that the implantation of an acellular dermal matrix covering a silicone prosthesis reduced the rate of inflammation in rats [25].

Our analysis reported an initial reaction of the resident subcutaneous tissue to the membrane implantation. Indeed, a different degree of membrane architecture modification and a consequent fluid accumulation surrounding the ACMs were reported. On day 14, the membrane structure of ACM1 appeared unaltered, and longitudinal MRI results revealed that it is still present until day 30, indicating a slow reabsorption rate. Moreover, the fluid accumulation appeared almost completely reabsorbed, indicating the high biocompatibility of ACM1 with the surrounding tissue. The slow reabsorption rate of ACM1 could be due to the two layers with different thicknesses and porosity that constitute the membrane. Low reabsorption could be advantageous for materials used as breast prosthesis coating due to a higher possibility of breast prosthesis integration that could reduce the PPC formation. A slow degradation rate allows the material to maintain its mechanical structure during tissue regeneration [31]. Instead, the volume of ACM2 and ACM3 decreased about 70% from 7 to 14 days after implantation, indicating a faster reabsorption degree than ACM1. On the other hand, the fluid accumulation remains elevated (of about 75%), allowing the development of oedema and recruitment of inflammatory cells.

The integration between ACMs and subcutaneous tissue was evaluated by analysing the membrane colonisation by resident cells and collagen fibres matrix. While ACM2 and ACM3 resulted poorly infiltrated, ACM1 appeared highly colonised, and native collagen fibres spread within the membrane starting from day 14. As suggested by histological evaluation, the integration process of ACM3 was detectable from day 30, indicating a later migration of endogenous extra-cellular matrix components through the membrane compared to ACM1. These results are supported by previous *in vitro* experiments, in which ASCs culture with ACM3 appeared suffering. A different behaviour was observed for ACM2. At 30 days, the membrane was not detectable by MRI or histological analysis, suggesting a rapid degradation and absorption process. This phenomenon could be attributable to an immune-inflammatory response induced by oedema visible at day 14.

Finally, adipogenic biostimulation was also evaluated. Only ACM1 showed positive results at the last evaluated time point. Specifically, a deposition of adipose tissue inside the membrane was clearly visible. The newly formed adipocytes appeared surrounded by extra-cellular matrix components. Collagen fibres, which constitute the major matrix component, resulted positive for collagen type III antibody by immunohistochemical analysis. Moreover, the angiogenesis phenomenon was detectable, and new vessels were found inside the adipose tissue. Since mature adipose tissue is composed of multiple components (such as adipocytes, extra-cellular matrix, vessels) [32], our findings suggest a multiple functional biostimulation induced by ACM1, which stimulated the formation of new well-structured adipose tissue. Indeed, ECM is responsible for providing growth factors for signalling, immunological response and modulation of structure properties of the tissue leading to its restoration and maintaining homeostasis [33]. The activation of adipogenesis after ACMs implantation hypothesises that adipocytes could improve the interaction between membrane and tissue, favouring the regeneration and repair processes. Another aspect that positively influences the regeneration is the angiogenesis by which resident mesenchymal stem cells could migrate into the damage site with growth factors and cytokines. In different models and clinical protocols, the implant of ACMs restores the physiologic vascularisation [34]. The stimulation of adipogenesis and the restoration of physiologic angiogenesis could ensure a *restitution ad integrum* of the dermis and subcutis [34].

In conclusion, this study evaluated three acellular matrices to be used as possible coating of breast silicone prosthesis. The results have shown that subcutaneously implanted natural membranes can induce biological activation in the surrounding tissue. Specifically, the induction of adipogenic stimulation is morphological-dependent on the porosity, thickness and swelling ratio of the membrane. The deposition of newly formed adipose tissue could play a key role in forming a well-organised tissue architecture preventing capsular contracture formation.
